# Cervical Cancer Prophylaxis—State-of-the-Art and Perspectives

**DOI:** 10.3390/healthcare10071325

**Published:** 2022-07-17

**Authors:** Patryk Poniewierza, Grzegorz Panek

**Affiliations:** 1 Medicover SP ZOO Company, Aleje Jerozolimskie 96, 00-807 Warsaw, Poland; 2 Department of Oncologic Gynecology and Obstetrics, The Center of Postgraduate Medical Education, 00-416 Warsaw, Poland; gmpanek@wp.pl

**Keywords:** cervical cancer, prevention, screening, vaccination, HPV, COVID-19, biomarkers, nanotheranostics

## Abstract

Background: Each year 604,127 new cases of cervical cancer (CC) are diagnosed, and 341,831 individuals die from the disease. It is the fourth most common cancer among women and the fourth most common cause of death from female cancers worldwide. The pathogenesis of CC is associated with human papillomavirus (HPV) infections and consists of several steps involving cell proliferation outside the human body’s control mechanisms. Strategies to prevent CC are based on screening and vaccination. Scope of the Review: The aim of this paper was to collect and analyze the available literature on the issue of CC prevention and the impact of the COVID-19 pandemic on its implementation. For this purpose, PubMed and Google Scholar databases were searched using keywords, such as “cervical cancer”; “HPV”; “prevention”; “prophylaxis”; “vaccination”; “screening” and “COVID-19” in different variations. Only articles published since 2018 were included in the study. Conclusions: Selected European countries have different CC prevention programs funded by national budgets. This translates into observed differences in the risk of death from CC (age-standardized rate Malta = 1.1, Poland = 5.9). COVID-19 pandemic due to disruption of CC screening may exacerbate these differences in the future. To improve the situation, new screening methods, such as p16/Ki67, HPV self-testing, and the use of artificial intelligence in colposcopic assessment, should be disseminated, as well as free HPV vaccination programs implemented in all countries. The search for new solutions is not without significance and entails ultra-sensitive screening tests for risk groups (mRNA E6/E7, SOX1/SOX14), HPV vaccines with shorter dosing schedules, and new therapeutic pathways using nanotheranostics.

## 1. Introduction

Each year 604,127 new cases of cervical cancer (CC) are diagnosed and 341,831 individuals die from the disease. It is the fourth most common cancer among women and the fourth most common cause of death from female cancers worldwide [1]. Although almost 85% of CC diagnoses worldwide are in developing countries, there are still 30,447 new cases each year in Europe. The incidence of CC in Europe varies, with the highest rates observed in Romania (age-standardized rate per 100,000 = 32.3) and the lowest in Malta (age-standardized rate per 100,000 = 5.7). Poland ranks eighth for incidence of CC and fifth for deaths. The five-year survival rate in Poland is 48.3% (the EU average is 62.1%) [2].

The pathogenesis of CC is associated with human papillomavirus (HPV) infections and consists of several steps involving cell proliferation outside the human body’s control mechanisms. This process results in a cascade of malignantly transformed cells in the following order: hyperplasia, dysplasia, carcinoma in situ, and invasive carcinoma [3]. HPV invades squamous cells, allowing the virus to continue to replicate and continuously infect cervical epithelial cells. The time required for invasive cancers to develop is a minimum of 10–12 years minimum. The most common type is HPV 16 (50% of cases). Other oncogenic HPV types responsible for persistent cervical tissue infections include HPV 18, 31, 33, 35, 39, 45, 51, 52, 56, 58, 59, 66, and 68. However, infection alone is not sufficient for disease development. Most infections are transient and resolve within a maximum of 2 years [4]. Strategies to prevent CC are based on screening and vaccination. Screening can be performed with cervical cytology alone (Pap smear, or liquid-based cytology—LBC) repeated every 3 years, with HPV DNA testing alone repeated every 5 years, or co-testing, being a combination of cytology and HPV DNA testing. HPV vaccination is recommended for girls and boys aged 9–14 years in two- or three-dose regimens [5,6]. These solutions have become the goals of strategies to eliminate CC as a global public health problem. The World Health Organization (WHO) has called for interventions to cover 70% of the population with CC screening using the HPV DNA test to vaccinate 90% of the population against HPV, and to enable access to CC diagnosis and treatment for 90% of women by 2030 [7]. Meeting these challenges requires defining the current state of knowledge in CC prevention and setting a course of action for the future considering the impact of additional factors, such as the COVID-19 pandemic. 

## 2. Aim of the Review

The aim of this paper was to collect and analyze the available literature on the issue of CC prevention and the impact of the COVID-19 pandemic on its implementation. For this purpose, PubMed and Google Scholar databases were searched using keywords, such as “cervical cancer”; “HPV”; “prevention”; “prophylaxis”; “vaccination”; “screening”, and “COVID-19” in different variations. Only articles published since 2018 were included in the study.

## 3. Review Results

### 3.1. Cervical Cancer Oncogenesis—Perspectives in Surveillance Programs

HPV oncoproteins E6 and E7, which prevent tumor suppressor genes from functioning properly by disrupting cell cycle checkpoints, play a key role in CC oncogenesis. The E6 protein degrades p53, which allows cells to continue replicating. The E7 protein causes an early entry into the S phase of the cell cycle through the degradation of pRb [8]. The above mechanisms of action prompt the utility of E6/E7 proteins as potential biomarkers. Currently, the most widely utilized screening tests use HPV DNA and the expression of p16 protein to directly determine the HPV oncogenic risk as well as using Ki-67, a protein responsible for proliferation. Despite the significant progress that has been made in the screening and treatment of CC, it is still necessary to search for new reliable screening methods [9]. 

Results from a study published in 2021 of 40,509 women showed that E6/E7 mRNA detection had the highest sensitivity among conventional cytology and p16/Ki-67 testing. Considering these findings, the E6/E7 mRNA assay appears to be a very good candidate for ultra-sensitive screening. The scientific consensus on the optimal sensitivity of tests used in CC screening remains an open question [10,11]. Potential highly sensitive CC biomarkers include SOX14. It belongs to a group of genes that are involved in the binding of the high-mobility group (HMG) domains to DNA, which stimulates the differentiation process in the cell cycle. With respect to CC, SOX 14 potentiates cell proliferation and invasiveness. The detection of SOX 14 allowed for the differential diagnosis of precancerous lesions and CC with a sensitivity of 94.12% and specificity of 86.46% [12]. In addition to the examples described above, potential CC biomarkers have been very well studied in the literature and are summarized in Table 1.

The HPV oncoprotein E7 affects cell growth processes by interacting with histone deacetylases (HDACs). Under physiological conditions, HDACs remove acetyl residues that inhibit transcription [17]. Nevertheless, the HDACs of classes I-III are involved in CC as a result of their interactions with the p50 protein, which exhibits antitumor activity (class I), promotion of cell motility (class II), and response to oxidative stress (class III), respectively [18]. Current knowledge allows us to conclude that HDAC inhibitors (HDACi) through various cellular pathways lead to apoptosis and exhibits adequate anticancer activity. They can be used alone and in combination with other drugs. The use of combination therapies can result in comparable therapeutic effects with fewer side effects [19]. Among the numerous cellular pathways that E6/E7 oncoproteins interact with is the JAK/STAT pathway. Signaling in this pathway contributes to tumor progression and metastatic development. Inhibitors of the JAK/STAT pathway are an area of interest in currently ongoing clinical trials. In addition to the assessment of toxicity, which according to current scientific reports may be significant. It is important to focus on the assessment of the disease-free period and overall survival of patients with CC [20]. Another pathway important for CC progression is the deregulation of the Notch pathway. This signaling pathway is associated with epithelial cell differentiation. HPV, which is dependent on keratinocyte differentiation, directly interferes with this process. Most invasive CCs show cytoplasmic localization of Notch1, with Notch1 in the cell nucleus correlating with poorer treatment outcomes [21]. An interesting phenomenon associated with HPV infection is the translation of apolipoprotein B mRNA editing enzyme, catalytic (APOBEC) gene proteins, which promote a tendency for cell mutagenesis. The presence of E6/E7 proteins has been shown to promote the up-regulation of APOBEC gene protein production [22]. APOBEC gene-mediated mutagenesis may be a future predictor of lesion progression to invasive cancer. In addition to interfering with cell proliferation, HPV infections have an immunomodulatory effect. Natural killer (NK) cells are involved in recognizing and eliminating HPV-infected cells. An interesting phenomenon is the avoidance by CC tumor cells to the responses that NK cells are capable of causing. Among the numerous mechanisms, the one by which the secretion of cytokines necessary for NK cell activation is inhibited is noteworthy [23]. The ability of NK cells to eliminate CC cells represents a promising direction in the search for immunotherapy-based drugs. The literature in this area is limited at this time. However, it is important to note that the ability of cells to evade the immune response cited above may affect the efficacy of therapeutic solutions developed. The role of HPV in the development of CC is undeniable. Nevertheless, the human leukocyte antigen (HLA) locus, which is responsible for the genetic predisposition to CC, has drawn the attention of researchers. Several studies have found independent risk variants associated with the 6p21.3 HLA locus. The estimated hereditary incidence of CC oscillates around 7%. Studies in large populations of HPV-negative CC outcomes are needed to verify this data [24,25]. HPV negative CCs are characterized by a variety of clinical courses as well as pathologies. HPV-negative CCs are diagnosed statistically later than HPV-positive CCs and correlate with shorter survival. Apart from the diagnostic delay, molecular correlations have not been demonstrated to date [26].

In conclusion, the process of CC oncogenesis is a target for new methods of prevention, prognosis of its severity, and creation of targeted therapies. Biomarkers p16/Ki-67 are being used increasingly and incorporated into standard protocols for conducting CC screening. E6/E7 mRNA tests and SOX1/SOX14 detection may become the starting points for making recommendations using ultra-sensitive screening tests especially in at-risk groups. Genetic aspects of CC oncogenesis warrant attention to the 6p21.3 HLA locus associated with heritable CC incidence. The above correlation can be used to develop special surveillance programs in genetically predisposed individuals. The apoptosis of cancer cells is an area of interest in terms of developing new therapies. HDAC inhibitors in CC can be used in combination treatment giving a similar therapeutic effect with fewer side effects. The JAK/STAT and Notch signaling pathways are responsible for the process of progression to CC. In the future, they may represent a predictive biomarker of treatment response and course of CC, as well as a point of interest for targeted therapies.

### 3.2. Cervical Cancer Prevention—Current Practice and Future Directions

#### 3.2.1. Cervical Cancer Screening

Current tests used for CC screening include Pap smear-based cervical cytology, LBC, HPV DNA, and co-testing [27,28]. Pap smear-based cervical cytology has been around for more than half a century. Its newer form is LBC. During a Pap smear, cells taken from the cervix are smeared on a slide, while in LBC, they are washed and suspended in a preservative solution. The advantage of LBC is that the material can be used for additional testing for HPV DNA [29,30]. The sensitivity of the Pap test is estimated to be 30–87% and specificity to be 86%. The number of false negative results ranges from 25% to 50% [31]. For LBC, the sensitivity is 61–66% with a specificity of 82–90% [32,33]. The sensitivity of Pap cytology is low and should be supplemented with other diagnostic methods [34,35]. A study published by Phaliwong et al. in 2018 on 28,564 cases showed that the percentage of unsatisfactory Pap smears was significantly higher than LBC (52.3% vs. 40.5%) [36]. The opinion suggesting the need for the extensive use of LBC in CC screening instead of a Pap smear is very well established in the literature [37,38,39,40]. In contrast, there is no evidence to explain that LBC differs from Pap smear in their accuracy in detecting CIN2+ lesions [41]. Nevertheless, LBC is preferred over Pap smear as a method of monitoring patients after CC treatment. The diagnostic accuracy of LBC is 97.6% and that of a Pap smear is 79.16% (patient group study (n = 94), significance of difference between groups *p* < 0.001) [42]. Despite the described advantages, LBC is not widely used worldwide (especially in developing countries due to the higher unit cost of the test compared to a Pap smear) [43]. It is difficult to argue with this argument, but it is important to note that LBC can reduce false negative results, decreasing the incidence of invasive CC cancer in the population, which in turn will reduce treatment costs. According to estimates from the German health care market, every EUR 1 spent on an LBC instead of a Pap smear will result in a savings of EUR 170 over the lifetime of a patient undergoing screening [29].

HPV infection can also be detected with a viral DNA test. HPV DNA tests are more sensitive than cervical cytology, so they can be performed less frequently (every 5 years). For the standard comparison test, the sensitivity is 98.7% and the specificity is 96% [44,45]. The high sensitivity of HPV testing can lead to over-detection of false positives, which are results in which n HPV infection is present but will not lead to CC in the long term. This situation can lead to patient exposure to further diagnostic testing, increase health care costs, and affect a woman’s perceptions about the effectiveness of screening [46,47]. There is a need to develop CC screening schemes based on the clinical context, as well as international validation of HPV testing. An interesting direction of exploration is the use of biomarkers as additional differentiation tests after HPV DNA positivity [48].

There are currently 254 HPV molecular tests available with over 425 variants. They use several diagnostic methods, including polymerase chain reaction (PCR) with sequencing, restriction fragment length polymorphism (RFLP) analysis, and hybridization assays [49]. A validation system, the VALidation of HPV GENotyping Tests (VALGENT), was introduced to establish common diagnostic criteria and to allow comparison of test results between tests [50]. Arbyn et al. reviewed an updated list of HPV tests adequate for CC screening. They recommended hrHPV DNA tests for CC screening resulting from the above review, including HC2, GP5+/6+ PCR-EIA, Abbott RealTime, Alinity, Anyplex HR, Cobas 4800, PapilloCheck, Onclarity, HPV-Risk, and Xpert HPV [45]. With new diagnostic methods emerging, systematic validation of recommended tests for CC screening is needed [51,52,53]. The efficacy of HPV DNA testing was confirmed in the HPV FOr cerviCAL Cancer Trial (FOCAL), in which a group of women (n = 5537) was followed up for 10 years after a negative HPV screening result. In the study group, the risk of developing a CIN2+ lesion was less than 1% after a 10-year follow-up period. The observation of another group for 48 months (n = 9552) indicated a CIN2+ risk of 0.5% [54,55]. Therefore, HPV tests should be considered to be of high value in protecting against the occurrence of CC for up to 10 years. The current consensus based on the availability of screening suggests repeated negative HPV tests at intervals of every 5 years [56,57]. Based on the Swedish experience, the costs associated with a diagnosis based on HPV testing (n = 16,544) and cervical cytology (n = 13,799) were compared. The average cost of detecting one HSIL+ case was EUR 9600 using HPV testing and EUR 7,600 using classical cytology. The nominal cost was significantly higher. Another study predicting the cost-effectiveness of screening with hrHPV testing showed a decrease in the incremental cost-effectiveness ratio (ICER) of (USD −6.16) relative to conventional cervical cytology. Quality-adjusted life-years (QALYs) were 386.98 for cytology and 390.80 for hrHPV-based testing, respectively [58,59]. A combination of LBC and HPV testing methods is called co-testing. Results from several randomized trials indicate an increase in the detection of precancerous lesions and a decrease in CC diagnoses after negative screening using these methods alone. The proportion of co-tests performed in one US state increased from 5.6% (2008) to 84.3% in women aged 30–64 years [60]. Interesting results were provided by a study in the pilot project “WOLPHSCREEN” on a group of 26,624 women, where it was shown that the incidence of CC significantly decreases when screening with co-testing is performed every 5 years [61]. The trend toward complex screening tests is also evident in the context of cytology itself, an extension of which is the p16/Ki67 dual stain cytology test (DSCT) with similar sensitivity but higher specificity. This method can significantly reduce the number of colposcopic examinations performed and protect patients from over-diagnosis while reducing costs [62,63]. In conclusion, despite many years of experience in the use of CC screening tests, there is still no clear consensus on the choice of a single method that combines accessibility, diagnostic performance, and cost-effectiveness. The use of artificial intelligence in screening methods can reduce costs by reducing the need for medical personnel, shorten the time needed for test results, as well as improve its accuracy by eliminating subjective evaluations by humans. Current research concludes that artificial intelligence can be used in both HPV testing and cervical cytology [64].

#### 3.2.2. Human Papillomavirus Vaccination

Current HPV vaccines include Gardasil^®^ (against HPV6, HPV11, HPV16, and HPV18 genotypes), Cervarix^®^ (against HPV16 and HPV 18 genotypes), and Gardasil 9^®^ (against HPV6, HPV11, HPV16, HPV18, HPV31, HPV33, HPV45, HPV52, and HPV58 genotypes). Worldwide, at least one dose has been administered to nearly 118 million women [65]. Global coverage of the complete vaccination schedule is estimated at 15%, and 40% for high-income countries. In Europe, population coverage with complete vaccination is estimated at 35% [66]. Countries that do not have a centrally funded national HPV vaccination program deviate significantly from the average. An example of such a country could be Poland, where vaccination is carried out locally using the budgets of local government units. Analysis of data from local government programs suggests a population coverage of girls aged 10–14 years of 2.05% [67]. Ninety percent of global CC deaths occur in low- and middle-income countries, which face difficulties in implementing effective prevention programs due to financial shortages and low public awareness. It is important to note, however, that the situations in these countries should improve from year to year. As of mid-2020, 41% of all low- and middle-income countries had initiated national HPV vaccination programs [68]. The spread of vaccination in highly developed countries faces a number of barriers such as the cost of the vaccine (in the absence of a national vaccination program), the lack of information about the HPV vaccine, the difficulty in completing the full series of vaccination, and the activities of anti-vaccination movements [69]. It is expected that as a result of the COVID-19 pandemic, the aforementioned barriers to HPV vaccination will increase. The recession in many countries caused by the COVID-19 pandemic may reduce subsidies supporting HPV vaccination programs in low- and middle-income countries. The supply chain disruption may have affected the vaccine production schedule, although there is no evidence of this. Anti-vaccination movements that gained impact during the COVID-19 pandemic spread false information discouraging vaccinations, including HPV [70]. Interesting results were provided by a study published by Smolarczyk et al. conducted on a group of 288 parents from Poland, in which 62.5% of the selected group said they had never heard of HPV. The level of knowledge of this group should be considered deeply inadequate, as only 49.4% gave correct answers to related questions [71]. Overcoming already known barriers can bring the goal of eliminating CC as a global public health problem closer. It is also necessary to set directions for further development. Ongoing research targets new dosing regimens of currently used vaccines (PATRICIA trial, which demonstrated the efficacy of a single dose of vaccine in preventing high-risk HPV infection) and new vaccines (the RG1-VLP vaccine provides broader coverage of HPV genotypes and is cheaper to produce) [72].

#### 3.2.3. Cervical Cancer Prevention Programs in Europe

The beginnings of organized screening dates back to 1968, when Wilson and Jungner developed a catalog of criteria that a disease must have in order to be screened. In Europe, the key date for CC screening was 1993 with the publication of the European Guidelines for Quality Assurance in Cervical Cancer Screening, which set out the principles for CC screening. Another edition issued in 2015 precisely formulated the criteria that a screening program must meet, i.e., the age of the target group, the time intervals for receiving the screening test, and the algorithm for further management depending on the results [73]. Table 2 shows the current information on the implementation of CC prevention programs in selected European countries.

For both screening and HPV vaccination programs, there is no uniform regimen across Europe. Most of the countries listed base CC screening on Pap smears at intervals of every 3 years. The exception is Malta, which has included LBC in its screening program. HPV testing is applied in Belgium, Denmark, and some regions of Portugal. Of the European countries, the only country with full replacement of cytology in favor of HPV testing in the Netherlands. A program based on HPV testing has been launched in the country in 2017. Samples are collected by primary care physicians (GPs) and if women do not enroll, samples for self-testing are sent out [81]. Almost all countries have an HPV vaccination program. The exception is Poland, which is the only country without such a solution. In German-speaking countries (Austria and Germany), boys are vaccinated in addition to girls. Differences in approaches to CC prevention are reflected in the mortality rates presented in each country. Based on data published by the International Agency for Research on Cancer, European countries were divided by their CC mortality rates, and the results are shown in Table 3 [89].

The highest risk of death from CC among the countries shown is in Poland. The lack of a national HPV vaccination program is telling. The lowest risk of death among countries is in Malta, which, in addition to its HPV vaccination program, applies LBC-based screening. It seems reasonable to standardize the recommendations for CC prevention programs funded by budgets in European countries. There is an urgent need to implement HPV vaccination funding in countries like Poland. The reasons for the success of the prevention program implemented in Malta need to be evaluated in further studies.

### 3.3. Cervical Cancer—The Impact of the COVID-19 Pandemic

The COVID-19 pandemic has significantly affected the level of implementation of CC prevention. In the first few months after the pandemic outbreak, CC screening completion dropped by 90%, and HPV vaccination completion dropped by more than 70% (March 2020) compared to the pre-pandemic period. Removal of lockdown restrictions improved CC prevention implementation only slightly. Compared to the pre-pandemic period, 35% decrease in screening and 25–50% decrease in HPV vaccination implementation (June 2020) were observed [90]. The decrease in screening performed may result in decreased detection of precancerous CC conditions and lead to more invasive cancers in the future. This impact, due to the small time period that has elapsed since the onset of the pandemic, is difficult to prove with retrospective data [91]. Estimates from models simulating screening in four countries (USA, Norway, Netherlands, Australia) suggest a 5–6% increase in CC cases [92]. A study by Burger et al. found that a six-month delay in screening carried out with Pap smear would lead to an increase in CC incidence by 5–7 cases per million women screened, while with co-tests this number would be slightly lower, estimated at 4–5 cases. A delay of two years results in an increase of 38–45 cases (Pap smear) and 35–45 cases (co-Test) per million women screened, respectively. Between 58–79% of the estimated CC cases occurred due to delay in further diagnosis and treatment, not in deferring the screening test itself [93]. Closing the gap in CC prevention due to COVID-19 pandemic must include prioritizing completion of outstanding screening tests and providing access to in-depth diagnosis and treatment for individuals with suspected or diagnosed CC. Particular attention should be paid to low- and middle-income countries (LMICs), in which 85% of worldwide CC cases are diagnosed. In these countries, CC prevention and access to treatment should be considered as inadequate also in the pre-pandemic period [94]. COVID-19 forced the introduction of lockdowns by state governments, impacted the financial crisis, disrupted supply chains, relocated medical worker resources to combat the pandemic, and limited the number of available medical facilities (creating monoprofile hospitals for COVID-19 patients). These actions have had a limiting effect on the prevention, diagnosis, and treatment of CC. Based on data from India, it is estimated that the population there suffered an increase of 16,808–50,035 disability adjusted life-years (DALYs) due to delay or discontinuation of CC treatment [95]. The COVID-19 pandemic, despite its negative impact on CC prevention, has created an impetus to seek new solutions and revise the approaches used. One method that has significantly gained in importance is self-testing. The self-testing method for hrHPV has a higher sensitivity than Pap smear and has similar accuracy to the HPV test collected by medical personnel [96]. Self-testing allows screening to be performed in areas of low population coverage, allows to compensate for differences created during the pandemic, and does not involve medical personnel who can be delegated to other tasks. The results of the CoCoss-Trial, involving 65 women in Germany, showed that a stand-alone test using a urine sample may be an alternative to self-testing for HPV [97]. The COVID-19 pandemic resulted in a change in the standards of practice for CC screening in favor of popularizing the use of LBCs, co-tests, or p16/Ki67. The Polish Society of Obstetricians and Gynecologists guidelines published at the end of 2021 additionally allowed the use of artificial intelligence (AI) algorithms for automated epithelial assessment after visual inspection after acetic acid (VIA) [98]. AI studies show that AI-enabled colposcopy improves the sensitivity of CIN2+ lesion detection [99]. Methods using decision support systems can be used in LMICs where the process of training medical personnel is significantly limited. The COVID-19 pandemic period due to lockdowns contributed to the development of telemedicine care. Telecolposcopy and digital colposcopy provides the ability to perform colposcopy in a variety of settings and transmit data for consultation of treatment decisions. The above strategy ensures that continuity of care is maintained as well as counteracts the concentration of patients requiring consultation at referral centers [100]. The constraints and impediments of the COVID-19 pandemic are forcing the development of new diagnostics and treatments—including precision therapies. Treatments using molecular and genetic profiling allow medical staff to focus their attention on high-risk groups, which is extremely important given limited staff resources. Precision therapy and diagnostics may also reduce the financial burden of CC prevention programs in the future [101]. Here, special attention should be paid to the emerging concept of nanotheranostics. This concept aims to combine cancer therapy and diagnosis within personalized medicine increasing survival and quality of life for cancer patients [102]. An example of a nanotheranostic concept is the use of tetraphenylethylene-based di-Pt(II) organometallic precursor (TPE-Pt), which is a chemotherapeutic with additional properties of a radiosensitizer, sensitizing cancer cells to radiotherapy. In vitro and in vivo studies in mice showed effective inhibition of tumor growth and prolonged survival of mice with cisplatin-resistant cancer [103]. In the context of CC treatment, there are promising reports using nanoparticles and small interfering RNA (siRNA), showing the ability to inhibit genes responsible for CC invasion and development [104]. The COVID-19 pandemic has significantly affected the financial, logistical, and social health of states, resulting in the disrupted implementation of CC prevention and treatment. At the same time, the advances in health care made during the pandemic forged new directions.

## 4. Conclusions

The presented arguments indicate that the path to the complete elimination of CC as a global public health problem by 2030 may prove extremely difficult:

(a) No uniform standards of CC prevention for European countries;
(b) Lack of implementation of a nationally funded HPV vaccination program in some European countries (e.g., Poland);
(c) Differences in risk of death from CC between European countries;
(d) Continued widening of inequalities in CC prevention delivery due to disruptions caused by the COVID-19 pandemic.

In conclusion, we would also like to highlight possible future directions to improve the implementation of CC prevention:

(a) The dissemination of new screening methods, such as LBC, HPV-Tests, p16/Ki67, self-testing, and use of AI in colposcopic evaluation;
(b) The acceleration of the evaluation of the clinical utility of new diagnostics, e.g., ultra-sensitive E6/E7 mRNA assays, SOX1/SOX14, and new therapeutic pathways using JAK/STAT signaling pathways, Notch, or the concept of nanotheranostics;
(c) The development of HPV vaccines with broader HPV genotype coverage and shorter dosing schedules.

## Figures and Tables

**Table 1:** Selected potential biomarkers of cervical cancer.

Potential Biomarker of Cervical Cancer	Sensitivity/Specificity [%]	Main Conclusion	Reference
SEPT9	89.5/63.3%	SEPT9 may be a potential screening and therapeutic biomarker in CC	Jiao et al. (2019) [13]
ZNF582	71/81%	The use of HPV DNA testing and the ZNF582 methylation assay improves diagnostic accuracy compared to HPV DNA testing alone.	Li et al. (2019) [14]
PAX1	86/85%	Diagnosis of PAX1 methylation can be incorporated into a CC screening regimen	Fang et al. (2019) [15]
SOX1	96/99%	The sensitivity and specificity of SOX1 allow its use in early detection programs for CC	Zhang et al. (2020) [16]

**Table 2:** Implementation of CC prevention programs in selected European countries.

Country	National CC Screening Program	National HPV Vaccination Program	Reference
Austria	Pap smear after age 18	Yes, for girls and boys	Sroczynski et al. (2020) [74]
Belgium	Pap smear every 3 years, possibility of HPV DNA test	Yes	Jolidon et al. (2020) [75]
Czech Republic	Pap smear every year over age 15	Yes	Altova et al. (2021) [76] Záhumenský et al. (2020) [77]
Denmark	Pap smear in women 23–49 years every 3 years 50–59 years every 5 years HPV test In women aged 60–64 years—once	Yes, girls 12 years old and older	Pedersen et al. (2018) [78]
Estonia	Pap smear every 5 years in women aged 30–55 years	Yes, girls aged 12–14	Ojamaa et al. (2018) [79]
France	Pap smear every 3 years in women aged 25–65 years	Yes, girls aged 11–14 Additional opportunity to vaccinate girls aged 15–19	de Rycke et al. (2020) [80]
The Netherlands	HPV test in women aged 30–60 years (65 years if previous HPV test was positive) at intervals of every 5 years until age 40, then every 10 years	Yes, vaccination of girls at age 12	Maver et al. (2020) [81] de Munter et al. (2021) [82]
Malta	LBC in women aged 27–39, Pap smear in women aged 40–64	Yes, girls at age 12	Deguara et al. (2021) [83]
Germany	Pap smear up to 35 years old, co-test 35–65 years old	Yes, for boys and girls aged 9–14	Hrgovic et al. (2020) [84] Wojcinski (2021) [85]
Poland	Pap smear in women aged 25–59, every 3 years	No	Osowiecka et al. (2021) [86]
Portugal	Determined by individual regions of the country (HPV test, LBC, Pap smear) performed every 3 years, or every 5 years in age groups 25–60, 25–64, 30–65	Yes, girls up to age 13	Marques et al. (2022) [87] Fernandes et al. (2022) [88]

**Table 3:** Cervical cancer—mortality rates in selected European countries.

Country	Age-Standardized Rate	Cumulative Mortality Risk
Austria	1.8	0.37
Belgium	2.0	0.42
Czech Republic	3.6	0.73
Denmark	2.2	0.53
Estonia	4.3	0.81
The Netherlands	1.4	0.32
France	2.2	0.42
Malta	1.1	0.26
Germany	2.2	0.44
Poland	5.9	1.04
Portugal	3.2	0.63

Age-standardized rate—per 100,000 women per year. Cumulative mortality risk—describes the probability of death between ages 0 and 74.

## Data Availability

International Agency for Research on Cancer Available online: https://gco.iarc.fr/today (accessed on 30 May 2022).

## References

[1] Rajaram S., Gupta B. (2021). Screening for cervical cancer: Choices & dilemmas. Indian J. Med. Res..

[2] Wierzba W., Jankowski M., Placiszewski K., Ciompa P., Jakimiuk A.J., Danska-Bidzinska A. (2021). Overall survival (OS) in patients after chemotherapy for cervical cancer in Poland in years 2008–2015. Ginekol. Pol..

[3] Martínez-Rodríguez F., Limones-González J.E., Mendoza-Almanza B., Esparza-Ibarra E.L., Gallegos-Flores P.I., Ayala-Luján J.L., Godina-González S., Salinas E., Mendoza-Almanza G. (2021). Understanding Cervical Cancer through Proteomics. Cells.

[4] Cooper D.B., McCathran C.E. (2022). Cervical Dysplasia. 2021 Dec. 16. StatPearls.

[5] Olusola P., Banerjee H.N., Philley J.V., Dasgupta S. (2019). Human Papilloma Virus-Associated Cervical Cancer and Health Disparities. Cells.

[6] Zhang S., Batur P. (2019). Human papillomavirus in 2019: An update on cervical cancer prevention and screening guidelines. Clevel. Clin. J. Med..

[7] Canfell K., Kim J.J., Brisson M., Keane A., Simms K.T., Caruana M., Burger E.A., Martin D., Nguyen D.T.N., Bénard E. (2020). Mortality impact of achieving WHO cervical cancer elimination targets: A comparative modelling analysis in 78 low-income and lower-middle-income countries. Lancet.

[8] Balasubramaniam S.D., Balakrishnan V., Oon C.E., Kaur G. (2019). Key Molecular Events in Cervical Cancer Development. Medicina.

[9] Volkova L.V., Pashov A.I., Omelchuk N.N. (2021). Cervical Carcinoma: Oncobiology and Biomarkers. Int. J. Mol. Sci..

[10] Giorgi Rossi P., Carozzi F., Ronco G., Allia E., Bisanzi S., Gillio-Tos A., De Marco L., Rizzolo R., Gustinucci D., Del Mistro A. (2021). p16/ki67 and E6/E7 mRNA Accuracy and Prognostic Value in Triaging HPV DNA-Positive Women. J. Natl. Cancer Inst..

[11] Sehnal B., Sláma J. (2020). What next in cervical cancer screening?. Ceska Gynekol..

[12] Zhao J., Cao H., Zhang W., Fan Y., Shi S., Wang R. (2021). SOX14 hypermethylation as a tumour biomarker in cervical cancer. BMC Cancer.

[13] Jiao X., Zhang S., Jiao J., Zhang T., Qu W., Muloye G.M., Kong B., Zhang Q., Cui B. (2019). Promoter methylation of SEPT9 as a potential biomarker for early detection of cervical cancer and its overexpression predicts radioresistance. Clin. Epigenet..

[14] Li N., He Y., Mi P., Hu Y. (2019). ZNF582 methylation as a potential biomarker to predict cervical intraepithelial neoplasia type III/worse: A meta-analysis of related studies in Chinese population. Medicine.

[15] Fang C., Wang S.Y., Liou Y.L., Chen M.H., Ouyang W., Duan K.M. (2019). The promising role of PAX1 (aliases: HUP48, OFC2) gene methylation in cancer screening. Mol. Genet. Genom. Med..

[16] Zhang L., Yu J., Huang W., Zhang H., Xu J., Cai H. (2020). A Sensitive and Simplified Classifier of Cervical Lesions Based on a Methylation-Specific PCR Assay: A Chinese Cohort Study. Cancer Manag. Res..

[17] Pešut E., Đukić A., Lulić L., Skelin J., Šimić I., Gašperov N.M., Tomaić V., Sabol I., Grce M. (2021). Human Papillomaviruses-Associated Cancers: An Update of Current Knowledge. Viruses.

[18] Liu S., Chang W., Jin Y., Feng C., Wu S., He J., Xu T. (2019). The function of histone acetylation in cervical cancer development. Biosci. Rep..

[19] Lourenço de Freitas N., Deberaldini M.G., Gomes D., Pavan A.R., Sousa A., Dos Santos J.L., Soares C.P. (2021). Histone Deacetylase Inhibitors as Therapeutic Interventions on Cervical Cancer Induced by Human Papillomavirus. Front. Cell Dev. Biol..

[20] Gutiérrez-Hoya A., Soto-Cruz I. (2020). Role of the JAK/STAT Pathway in Cervical Cancer: Its Relationship with HPV E6/E7 Oncoproteins. Cells.

[21] Rodrigues C., Joy L.R., Sachithanandan S.P., Krishna S. (2019). Notch signalling in cervical cancer. Exp. Cell Res..

[22] Revathidevi S., Murugan A.K., Nakaoka H., Inoue I., Munirajan A.K. (2021). APOBEC: A molecular driver in cervical cancer pathogenesis. Cancer Lett..

[23] Gutiérrez-Hoya A., Soto-Cruz I. (2021). NK Cell Regulation in Cervical Cancer and Strategies for Immunotherapy. Cells.

[24] Ramachandran D., Schürmann P., Mao Q., Wang Y., Bretschneider L.M., Speith L.M., Hülse F., Enßen J., Bousset K., Jentschke M. (2020). Association of genomic variants at the human leukocyte antigen locus with cervical cancer risk, HPV status and gene expression levels. Int. J. Cancer.

[25] Ramachandran D., Dörk T. (2021). Genomic Risk Factors for Cervical Cancer. Cancers.

[26] Yoshida H., Shiraishi K., Kato T. (2021). Molecular Pathology of Human Papilloma Virus-Negative Cervical Cancers. Cancers.

[27] Shami S., Coombs J. (2021). Cervical cancer screening guidelines. J. Am. Acad. Physician Assist..

[28] Hussain E., Mahanta L.B., Borah H., Das C.R. (2020). Liquid based-cytology Pap smear dataset for automated multi-class diagnosis of pre-cancerous and cervical cancer lesions. Data Brief.

[29] Armstrong S.F., Guest J.F. (2020). Cost-Effectiveness and Cost-Benefit of Cervical Cancer Screening with Liquid Based Cytology Compared with Conventional Cytology in Germany. Clin. Outcomes Res..

[30] Zhang S., Xu H., Zhang L., Qiao Y. (2020). Cervical cancer: Epidemiology, risk factors and screening. Chin. J. Cancer Res..

[31] Kamineni V., Nair P., Deshpande A. (2019). Can LBC Completely Replace Conventional Pap Smear in Developing Countries. J. Obstet. Gynecol. India.

[32] Bogdanova A., Andrawos C., Constantinou C. (2022). Cervical cancer, geographical inequalities, prevention and barriers in resource depleted countries (Review). Oncol. Lett..

[33] Najib F.S., Hashemi M., Shiravani Z., Poordast T., Sharifi S., Askary E. (2020). Diagnostic Accuracy of Cervical Pap Smear and Colposcopy in Detecting Premalignant and Malignant Lesions of Cervix. Indian J. Surg. Oncol..

[34] Pankaj S., Nazneen S., Kumari S., Kumari A., Kumari A., Kumari J., Choudhary V., Kumar S. (2018). Comparison of conventional Pap smear and liquid-based cytology: A study of cervical cancer screening at a tertiary care center in Bihar. Indian J. Cancer.

[35] Nkwabong E., Badjan I.L.B., Sando Z. (2018). Pap smear accuracy for the diagnosis of cervical precancerous lesions. Trop. Dr..

[36] Phaliwong P., Pariyawateekul P., Khuakoonratt N., Sirichai W., Bhamarapravatana K., Suwannarurk K. (2018). Cervical Cancer Detection between Conventional and Liquid Based Cervical Cytology: A 6-Year Experience in Northern Bangkok Thailand. Asian Pac. J. Cancer Prev..

[37] Hashmi A.A., Naz S., Ahmed O., Yaqeen S.R., Irfan M., Asif M.G., Kamal A., Faridi N. (2020). Comparison of Liquid-Based Cytology and Conventional Papanicolaou Smear for Cervical Cancer Screening: An Experience From Pakistan. Cureus.

[38] Gupta R., Yadav R., Sharda A., Kumar D., Sandeep, Mehrotra R., Gupta S. (2019). Comparative evaluation of conventional cytology and a low-cost liquid-based cytology technique, EziPREP™, for cervicovaginal smear reporting: A split sample study. Cytojournal.

[39] Shobana R., Saranya B. (2019). Comparison of conventional Papanicolaou smear and liquid-based cytology for cervical cancer screening. Int. J. Sci. Study.

[40] Krishna C., Chandraiah S., Krishna C. (2021). Comparison of conventional Papanicolaou smear and liquid-based cytology: A study of cervical cancer screening at a tertiary care centre in Bengaluru. Int. J. Reprod. Contracept. Obstet. Gynecol..

[41] Hillemanns P., Friese K., Dannecker C., Klug S., Seifert U., Iftner T., Hädicke J., Löning T., Horn L., Schmidt D. (2019). Prevention of Cervical Cancer: Guideline of the DGGG and the DKG (S3 Level, AWMF Register Number 015/027OL, December 2017)—Part 1 with Introduction, Screening and the Pathology of Cervical Dysplasia. Geburtshilfe Frauenheilkd.

[42] Singh U., Anjum, Qureshi S., Negi N., Singh N., Goel M., Srivastava K. (2018). Comparative study between liquid-based cytology & conventional Pap smear for cytological follow up of treated patients of cancer cervix. Indian J. Med. Res..

[43] Honarvar Z., Zarisfi Z., Salari Sedigh S., Masoumi Shahrbabak M. (2022). Comparison of conventional and liquid-based Pap smear methods in the diagnosis of precancerous cervical lesions. J. Obstet. Gynaecol..

[44] Perkins R.B., Guido R.L., Saraiya M., Sawaya G.F., Wentzensen N., Schiffman M., Feldman S. (2021). Summary of Current Guidelines for Cervical Cancer Screening and Management of Abnormal Test Results: 2016–2020. J. Women’s Health.

[45] Arbyn M., Simon M., Peeters E., Xu L., Meijer C.J.L.M., Berkhof J., Cuschieri K., Bonde J., Ostrbenk Vanlencak A., Zhao F.-H. (2021). 2020 list of human papillomavirus assays suitable for primary cervical cancer screening. Clin. Microbiol. Infect..

[46] Schiffman M., de Sanjose S. (2019). False positive cervical HPV screening test results. Papillomavirus Res..

[47] Veijalainen O., Kares S., Kotaniemi-Talonen L., Kujala P., Vuento R., Luukkaala T., Kholová I., Mäenpää J. (2021). Primary HPV screening for cervical cancer: Results after two screening rounds in a regional screening program in Finland. Acta Obstet. Gynecol. Scand..

[48] Johnson L.G., Saidu R., Svanholm-Barrie C., Boa R., Moodley J., Tergas A., Persing D., Campbell S.A., Tsai W.Y., Wright T.C. (2022). Clinical Utility of Reflex Testing with Cancer Biomarkers to Improve Diagnostic Accuracy of Primary Human Papillomavirus Screening. Cancer Epidemiol. Biomark. Prev..

[49] Chrysostomou A.C., Kostrikis L.G. (2020). Methodologies of Primary HPV Testing Currently Applied for Cervical Cancer Screening. Life.

[50] Heideman D.A.M., Xu L., Hesselink A.T., Doorn S., Ejegod D.M., Pedersen H., Quint W.G.V., Bonde J., Arbyn M. (2019). Clinical performance of the HPV-Risk assay on cervical samples in SurePath medium using the VALGENT-4 panel. J. Clin. Virol..

[51] Wei B., Mei P., Huang S., Yu X., Zhi T., Wang G., Xu X., Xiao L., Dong X., Cui W. (2020). Evaluation of the SureX HPV genotyping test for the detection of high-risk HPV in cervical cancer screening. Virol. J..

[52] Gustavsson I., Aarnio R., Myrnäs M., Hedlund-Lindberg J., Taku O., Meiring T., Wikström I., Enroth S., Williamson A.-L., Olovsson M. (2019). Clinical validation of the HPVIR high-risk HPV test on cervical samples according to the international guidelines for human papillomavirus DNA test requirements for cervical cancer screening. Virol. J..

[53] Demarco M., Carter-Pokras O., Hyun N., Castle P.E., He X., Dallal C.M., Chen J., Gage J.C., Befano B., Fetterman B. (2018). Validation of a Human Papillomavirus (HPV) DNA Cervical Screening Test That Provides Expanded HPV Typing. J. Clin. Microbiol..

[54] Gottschlich A., van Niekerk D., Smith L.W., Gondara L., Melinikow J., Cook D.A., Lee M., Stuart G., Martin R.E., Peacock S. (2021). Assessing 10-Year Safety of a Single Negative HPV Test for Cervical Cancer Screening: Evidence from FOCAL-DECADE Cohort. Cancer Epidemiol. Biomark. Prev..

[55] Ogilvie G.S., Van Niekerk D., Krajden M., Smith L.W., Cook D., Gondara L., Ceballos K., Quinlan D., Lee M., Martin R.E. (2018). Effect of Screening with Primary Cervical HPV Testing vs Cytology Testing on High-grade Cervical Intraepithelial Neoplasia at 48 Months: The HPV FOCAL Randomized Clinical Trial. JAMA.

[56] Ibáñez R., Roura E., Monfil L., Rodríguez L.A., Sardà M., Crespo N., Pascual A., Martí C., Fibla M., Gutiérrez C. (2020). Long-term protection of HPV test in women at risk of cervical cancer. PLoS ONE.

[57] Gilham C., Sargent A., Kitchener H.C., Peto J. (2019). HPV testing compared with routine cytology in cervical screening: Long-term follow-up of ARTISTIC RCT. Health Technol. Assess..

[58] Bergengren L., Ryen L., Flodström C., Fadl H., Udumyen R., Karlsson M.G., Helenius G. (2021). Effectiveness and costs of an implemented primary HPV cervical screening programme in Sweden—A population based cohort study. Prev. Med. Rep..

[59] Vale D.B., Silva M.T., Discacciati M.G., Polegatto I., Teixeira J.C., Zeferino L.C. (2021). Is the HPV-test more cost-effective than cytology in cervical cancer screening? An economic analysis from a middle-income country. PLoS ONE.

[60] Cuzick J., Du R., Adcock R., Kinney W., Joste N., McDonald R.M., English K., Torres S.M., Saslow D., Wheeler C.M. (2021). Uptake of co-testing with HPV and cytology for cervical screening: A population-based evaluation in the United States. Gynecol. Oncol..

[61] Horn J., Denecke A., Luyten A., Rothe B., Reinecke-Lüthge A., Mikolajczyk R., Petry K.U. (2019). Reduction of cervical cancer incidence within a primary HPV screening pilot project (WOLPHSCREEN) in Wolfsburg, Germany. Br. J. Cancer.

[62] Zhang R., Ge X., You K., Guo Y., Guo H., Wang Y., Geng L. (2018). p16/Ki67 dual staining improves the detection specificity of high-grade cervical lesions. J. Obstet. Gynaecol. Res..

[63] Areán-Cuns C., Mercado-Gutiérrez M., Paniello-Alastruey I., Mallor-Giménez F., Córdoba-Iturriagagoitia A., Lozano-Escario M., Santamaria-Martínez M. (2018). Dual staining for p16/Ki67 is a more specific test than cytology for triage of HPV-positive women. Virchows Arch..

[64] Hou X., Shen G., Zhou L., Li Y., Wang T., Ma X. (2022). Artificial Intelligence in Cervical Cancer Screening and Diagnosis. Front. Oncol..

[65] Wang R., Pan W., Jin L., Huang W., Li Y., Wu D., Gao C., Ma D., Liao S. (2020). Human papillomavirus vaccine against cervical cancer: Opportunity and challenge. Cancer Lett..

[66] Bruni L., Saura-Lázaro A., Montoliu A., Brotons M., Alemany L., Diallo M.S., Afsar O.Z., LaMontagne D.S., Mosina L., Contreras M. (2021). HPV vaccination introduction worldwide and WHO and UNICEF estimates of national HPV immunization coverage 2010–2019. Prev. Med..

[67] Augustynowicz A., Bojar I., Borowska M., Bobiński K., Czerw A. (2020). Self-government HPV vaccination programmes in Poland, 2009–2016. Ann. Agric. Environ. Med..

[68] Tsu V.D., LaMontagne D.S., Atuhebwe P., Bloem P.N., Ndiaye C. (2021). National implementation of HPV vaccination programs in low-resource countries: Lessons, challenges, and future prospects. Prev. Med..

[69] Oyedeji O., Maples J.M., Gregory S., Chamberlin S.M., Gatwood J.D., Wilson A.Q., Zite N.B., Kilgore L.C. (2021). Pharmacists’ Perceived Barriers to Human Papillomavirus (HPV) Vaccination: A Systematic Literature Review. Vaccines.

[70] Toh Z.Q., Russell F.M., Garland S.M., Mulholland E.K., Patton G., Licciardi P.V. (2021). Human Papillomavirus Vaccination After COVID-19. JNCI Cancer Spectr..

[71] Smolarczyk K., Duszewska A., Drozd S., Majewski S. (2022). Parents’ Knowledge and Attitude towards HPV and HPV Vaccination in Poland. Vaccines.

[72] Dilley S., Miller K.M., Huh W.K. (2020). Human papillomavirus vaccination: Ongoing challenges and future directions. Gynecol. Oncol..

[73] Chrysostomou A.C., Stylianou D.C., Constantinidou A., Kostrikis L.G. (2018). Cervical Cancer Screening Programs in Europe: The Transition Towards HPV Vaccination and Population-Based HPV Testing. Viruses.

[74] Sroczynski G., Esteban E., Widschwendter A., Oberaigner W., Borena W., Von Laer D., Hackl M., Endel G., Siebert U. (2020). Reducing overtreatment associated with overdiagnosis in cervical cancer screening—A model-based benefit–harm analysis for Austria. Int. J. Cancer.

[75] Jolidon V., De Prez V., Willems B., Bracke P., Cullati S., Burton-Jeangros C. (2020). Never and under cervical cancer screening in Switzerland and Belgium: Trends and inequalities. BMC Public Health.

[76] Altová A., Kulhánová I., Brůha L., Lustigová M. (2021). Breast and cervical cancer screening attendance among Czech women. Central Eur. J. Public Health.

[77] Záhumenský J., Pšenková P., Nadzámová A., Drabiščáková P., Hruban L., Weinberger V., Kacerovský M., Dosedla E. (2020). Comparison of opinions of Slovak and Czech female medical students on HPV vaccination. Central Eur. J. Public Health.

[78] Pedersen K., Fogelberg S., Thamsborg L.H., Clements M., Nygård M., Kristiansen I.S., Lynge E., Sparén P., Kim J.J., Burger E.A. (2018). An overview of cervical cancer epidemiology and prevention in Scandinavia. Acta Obstet. Gynecol. Scand..

[79] Ojamaa K., Innos K., Baburin A., Everaus H., Veerus P. (2018). Trends in cervical cancer incidence and survival in Estonia from 1995 to 2014. BMC Cancer.

[80] De Rycke Y., Tubach F., Lafourcade A., Guillo S., Dalichampt M., Dahlab A., Bresse X., Uhart M., Bergeron C., Borne H. (2020). Cervical cancer screening coverage, management of squamous intraepithelial lesions and related costs in France. PLoS ONE.

[81] Maver P.J., Poljak M. (2020). Primary HPV-based cervical cancer screening in Europe: Implementation status, challenges, and future plans. Clin. Microbiol. Infect..

[82] de Munter A.C., Klooster T.M.S.-V.T., van Lier A., Akkermans R., de Melker H.E., Ruijs W.L.M. (2021). Determinants of HPV-vaccination uptake and subgroups with a lower uptake in the Netherlands. BMC Public Health.

[83] Deguara M., Calleja N., England K. (2021). Cervical cancer and screening: Knowledge, awareness and attitudes of women in Malta. J. Prev. Med. Hyg..

[84] Hrgovic Z., Fures R., Jaska S. (2020). Implementation of the Program for Early Detection of Cervical Cancer in the Federal Republic of Germany. Mater. Socio Med..

[85] Osowiecka K., Yahuza S., Szwiec M., Gwara A., Kasprzycka K., Godawska M., Olejniczak D., Nowacka A., Nowakowski J.J., Nawrocki S. (2021). Students’ Knowledge about Cervical Cancer Prevention in Poland. Medicina.

[86] Wojcinski M. (2021). 14 Jahre HPV-Impfung: Was haben wir erreicht?. Gynakologe.

[87] Marques P., Geraldes M., Gama A., Heleno B., Dias S. (2022). Non-attendance in cervical cancer screening among migrant women in Portugal: A cross-sectional study. Women’s Health.

[88] Fernandes C., Alves J., Rodrigues A., Azevedo J. (2022). Group for the Study of HPV Vaccines. Epidemiological impact of the human papillomavirus vaccination program on genital warts in Portugal: A retrospective, chart review study. Vaccine.

[89] Ferlay J., Ervik M., Lam F., Colombet M., Mery L., Piñeros M., Znaor A., Soerjomataram I., Bray F. (2020). Global Cancer Observatory: Cancer Today.

[90] Smith D.L., Perkins R.B. (2022). Low rates of HPV vaccination and cervical cancer screening: Challenges and opportunities in the context of the COVID-19 pandemic. Prev. Med..

[91] Wentzensen N., Clarke M.A., Perkins R.B. (2021). Impact of COVID-19 on cervical cancer screening: Challenges and opportunities to improving resilience and reduce disparities. Prev. Med..

[92] Smith M.A., Burger E.A., Castanon A., de Kok I.M.C.M., Hanley S.J.B., Rebolj M., Hall M.T., Jansen E.E., Killen J., O’Farrell X. (2021). Impact of disruptions and recovery for established cervical screening programs across a range of high-income country program designs, using COVID-19 as an example: A modelled analysis. Prev. Med..

[93] Burger E.A., Jansen E.E., Killen J., de Kok I.M., Smith M.A., Sy S., Dunnewind N., Campos N.G., Haas J.S., Kobrin S. (2021). Impact of COVID-19-related care disruptions on cervical cancer screening in the United States. J. Med. Screen..

[94] Del Pilar Estevez-Diz M., Bonadio R.C., Miranda V.C., Carvalho J.P. (2020). Management of cervical cancer patients during the COVID-19 pandemic: A challenge for developing countries. Ecancermedicalscience.

[95] Gupta N., Chauhan A.S., Prinja S., Pandey A.K. (2021). Impact of COVID-19 on Outcomes for Patients With Cervical Cancer in India. JCO Glob. Oncol..

[96] Lozar T., Nagvekar R., Rohrer C., Dube Mandishora R.S., Ivanus U., Fitzpatrick M.B. (2021). Cervical Cancer Screening Postpandemic: Self-Sampling Opportunities to Accelerate the Elimination of Cervical Cancer. Int. J. Women’s Health.

[97] Ertik F.C., Kampers J., Hülse F., Stolte C., Böhmer G., Hillemanns P., Jentschke M. (2021). CoCoss-Trial: Concurrent Comparison of Self-Sampling Devices for HPV-Detection. Int. J. Environ. Res. Public Health.

[98] Zimmer M., Bidzinski M., Wielgos M. Cervical Cancer Screening—Recommendations of the Polish Society of GyneCologists and Obstetricians, 2021. https://ptgin.pl/node/82.

[99] Zhao Y., Li Y., Xing L., Lei H., Chen D., Tang C., Li X. (2022). The Performance of Artificial Intelligence in Cervical Colposcopy: A Retrospective Data Analysis. J. Oncol..

[100] Ciavattini A., Delli Carpini G., Giannella L., Arbyn M., Kyrgiou M., Joura E.A., Sehouli J., Carcopino X., Redman C.W., Nieminen P. (2020). European Federation for Colposcopy (EFC) and European Society of Gynaecological Oncology (ESGO) joint considerations about human papillomavirus (HPV) vaccination, screening programs, colposcopy, and surgery during and after the COVID-19 pandemic. Int. J. Gynecol. Cancer.

[101] Hu Z., Ma D. (2018). The precision prevention and therapy of HPV-related cervical cancer: New concepts and clinical implications. Cancer Med..

[102] Chen X., Zheng G., Cheng J., Yang Y.-Y. (2019). Supramolecular Nanotheranostics. Theranostics.

[103] Ding Y., Tong Z., Jin L., Ye B., Zhou J., Sun Z., Yang H., Hong L., Huang F., Wang W. (2022). An NIR Discrete Metallacycle Constructed from Perylene Bisimide and Tetraphenylethylene Fluorophores for Imaging-Guided Cancer Radio-Chemotherapy. Adv. Mater..

[104] Xia Y., Tang G., Wang C., Zhong J., Chen Y., Hua L., Li Y., Liu H., Zhu B. (2019). Functionalized selenium nanoparticles for targeted siRNA delivery silence Derlin1 and promote antitumor efficacy against cervical cancer. Drug Deliv..

